# Study on the impact of smart city construction on the health of the elderly population——A quasi-natural experiment in China

**DOI:** 10.1371/journal.pone.0305897

**Published:** 2024-06-21

**Authors:** Juqiu Deng, Dong Yao, Yue Deng, Zhenyu Liu, Jiayu Yang, Dezhao Gong

**Affiliations:** 1 School of Economics, Sichuan University, Chengdu, Sichuan, China; 2 Chengdu Jincheng College, Chengdu, Sichuan, China; 3 West China Hospital, Sichuan University/West China School of Nursing, Sichuan University, Chengdu, Sichuan, China; Zhongnan University of Economics and Law, CHINA

## Abstract

In the context of global aging, promoting the health of the elderly has become a critical issue. However, whether the development of smart cities can impact the health of older adults remains to be further validated. In this paper, based on panel data from the China Health and Retirement Longitudinal Study (CHARLS), a difference in difference model is used to empirically investigate whether smart city construction improves the health of older people in the region. The results show that smart city construction enhances the health of the elderly. Specifically, the construction achieved a significant improvement in the physical health of the elderly who did not live with their children. The health promotion effect of the smart city was more significant for the urban elderly than for the rural elderly. The elucidated mechanisms of influence suggest that smart cities bring about their effects through the promotion of urban leisure infrastructure, enhancement of medical service provision, advancement in urban environmental protection and stimulation of urban information and communication technology infrastructure development.

## 1. Introduction

Population aging is a major reality faced by nations worldwide, and China is undergoing the most extensive and fastest aging process on a global scale. According to the United Nations’ "World Population Prospects 2022" report, the proportion of the global population aged 65 and above is projected to increase from 10% to 16% between 2022 and 2050. Notably in 2020, China’s population aged 60 and above reached 260 million, accounting for 18.7% of the total population and projections indicate that by the year 2039, the proportion of the elderly population will reach 30% [1]. Therefore, China is anticipated to maintain the world’s largest elderly population in the coming years [2]. The observable aging trend in China is poised to substantially escalate the demand for healthcare services and living environment among the elderly, which imposes a more severe test on the capacity, resource allocation, and service quality of the governance system and the healthcare system. Consequently, it is imperative to elucidate the influencing factors of elderly health, explore pathways for enhancing the health status of the elderly, and establish a theoretical basis for measures aimed at improving elderly well-being and maintaining the stability of socioeconomic operations.

In the 2016 World Report on Aging and Health, the World Health Organization categorized the factors affecting "healthy aging" into intrinsic individual capacities and environmental characteristics. In detail, intrinsic individual capabilities, including income level, educational attainment, health insurance, social networks, and the state of retirement [3] have a direct impact on the health status of the elderly [4]. There is a strong correlation between the income and wealth of the elderly and their access to adequate medical services [5], while relative income levels have an impact on individual health through psychosocial factors [6,7]. Financial status also has an indirect impact on health through the provision of health insurance [8] and living conditions. The provision of health insurance fosters the utilisation of medical services, increasing total healthcare expenditures for the elderly, enhancing their health status, and mitigating family medical burdens [9]. Additionally, higher levels of education render it easier for the elderly to access health information, respond effectively to problems and choose healthy lifestyles [10,11]. Environmental characteristics, on the other hand, include all the external factors that influence an individual’s living environment. From a psychological health standpoint, social support and feelings of loneliness wield a significant impact on the health of the elderly. Typically, those with strong social networks and intimacy are generally healthier and happier [12], with family care complementing health services by reducing barriers to seeking medical care, enhancing the accessibility of healthcare access and addressing the increasing demand for medical services [13]. Besides, residential registration (hukou) [14]and the characteristics of residents’ areas, such as safety, adequate housing conditions and accessibility to community facilities also influence the health levels of the elderly [15]. In terms of geographic and demographic distribution, there are linkages between the spatial accessibility of healthcare and the health status of the elderly [16]. Specifically, high-density development will encroach on a large amount of natural resources such as arable land and green space and replace them with construction sites, leaving a lack of green space which will seriously harm the physical and mental health of the residents. This implies that the elderly require a high quality living environment.

With the acceleration of urbanisation, there is a growing concern about the relationship between the level of urban development and the health of the population. Importantly, In urban geography, it is emphasized that the level of urban development has a significant impact on health [17]. Health-promoting behaviours such as healthy diet, physical activity, and social interactions in cities can significantly contribute to elevating residents’ health levels. Spatial distribution and accessibility of shops and supermarkets have a significant impact on people’s diet and lifestyle [18–20]. Concurrently, the accessibility of open spaces such as streets, parks, squares, and green areas influences residents’ engagement in recreational activities [21–23], thereby impacting their overall health [24–26]. Hence, a robust association exists between urban residential density and residents’ health levels [24–26]. High-density development often represents relatively short commuting distances between housing, work, shopping and other destinations, which encourages physical activity such as walking [24,27]. Nevertheless, a high density of living space may induce a variety of environmental stresses, such as noise, pollution and overcrowding [28].

Health geography has long been concerned with the effects of these environmental factors on human health [29]. For example, air pollution, including PM2.5 and PM1, can affect the human nervous, respiratory, and cardiovascular systems, leading to a reduction in individual cognitive abilities [30]. Prolonged exposure to air pollution can increase psychological stress [31], depression, anxiety [32] and suicidal tendencies [33]. Noise pollution, comprising road traffic noise, rail transit noise, commercial and dining noise, and construction noise, negatively affects residents’ sleep quality, contributing to depression, anxiety, and diminished happiness [34]. Inadequate access to high-quality, safe drinking water hinders the control of bacterial and viral transmission, resulting in increased rates of respiratory and skin diseases, as well as diarrhea and infectious diseases [35,36]. Therefore, cities with high-quality air and a pedestrian-friendly street network are conducive to well-being [37,38]. The elderly population, in particular, emphasizes a greater need for advancements in urban development. This heightened demand is attributed to several factors. Firstly, environmental pollution exerts a more pronounced and adverse effect on the health of the elderly compared to healthier individuals and younger generations. Secondly, the unique health needs of the elderly demand a higher standard of development for urban medical systems to cater to their specific requirements adequately [39]. Lastly, the integration of information technology in geriatric health presents opportunities for reducing medical costs, enhancing healthcare quality, and boosting the health of the elderly [40], necessitating corresponding development in the information infrastructure of cities. The aforementioned viewpoints suggest that the level of urban development is an important factor in the well-being of the elderly.

Currently, smart cities are improving the living standards of residents by improving healthcare, education, transportation, and agriculture [41]. However, few studies have researched the health of the elderly in association with China’s ongoing Smart City Construction (SCC) and a systematic analysis of the impact mechanism of SCC on elderly health is lacking. To address this gap, this paper will concentrate on the health effects of SCC on the elderly and the underlying impact mechanisms and it aims to answer the following questions: First, can the implementation of SCC influence the health status of the elderly population? Second, what are the underlying impact mechanisms associated with the influence of smart cities on elderly health? Specifically, which domains of smart city construction exhibit significant effects in enhancing the health of the elderly? Third, given the vast regional development disparities in China, is the impact of SCC on the health of the older population universal? Does it vary due to factors such as urban-rural dichotomy and family structure? To answer these questions, this study examined the impact of SCC on the health of the elderly population based on panel data from the China Health and Retirement Longitudinal Study (CHARLS) from 2011 to 2018, utilizing a multi-temporal double-difference-in-differences (DID) model, and explores the potential impact mechanisms and heterogeneity of its effects.

The potential marginal contributions of this paper are as follows: (1) It expands the literature on the policy effects of smart city development. Previous research has extensively examined the impact of smart cities on urban efficiency and environmental governance but has not addressed the health of the elderly. This paper focuses on how smart cities specifically benefit this demographic. (2) It explores the mechanisms by which smart city development impacts elderly health. This study not only investigates the effects of smart city development on the health of the elderly but also comprehensively analyzes the pathways through which smart city development influences elderly health, including urban leisure infrastructure, healthcare service provision, and environmental protection. (3) It examines regional and population heterogeneity. The research considers the heterogeneity in the impact of smart city development on the health of the elderly across different regions and population groups in China. This includes studying the differences between urban and rural elderly populations as well as between elderly individuals with children and those without. This approach provides a deeper understanding of how the benefits of smart cities vary by location and family structure, which is crucial for decision-making.

## 2. Empirical research design

### 2.1 Policy background

The core concept of a smart city is to embed a variety of smart sensors in urban public resources (e.g. hospitals, oil and gas pipelines, highways, subways and buildings) and integrate them into an Internet of Things system that senses key information about the operation of the core urban system in real time. Through the use of cloud computing, big data and other new generation information technology, the data generated in the city’s operation will be analyzed and integrated in order to achieve accurate management and efficient allocation of urban resources. This concept originated from "Smart Earth" proposed by IBM in 2008, and formally evolved into "Smart City" in 2010 [42]. The concept of smart cities has attracted widespread attention around the world, especially in developed countries such as the United States, the United Kingdom, Japan and South Korea. Over the past decade, smart cities have become a common trend for cities around the world to pursue and develop in the information technology era, with more and more countries and regions joining the ranks of smart city planning and construction.

Compared with developed countries, China started late in smart city construction, showing a "point to face" process of innovation and diffusion. 2009, Guangzhou, Guangdong Province, launched smart city construction, and signed a cooperation program with IBM, marking the beginning of China’s smart city construction [43]. Subsequently, local governments such as Kunshan, Jiangsu Province and Wuhan, Hubei Province also signed cooperation agreements with IBM, creating a precedent for smart city construction.

**Fig 1:**
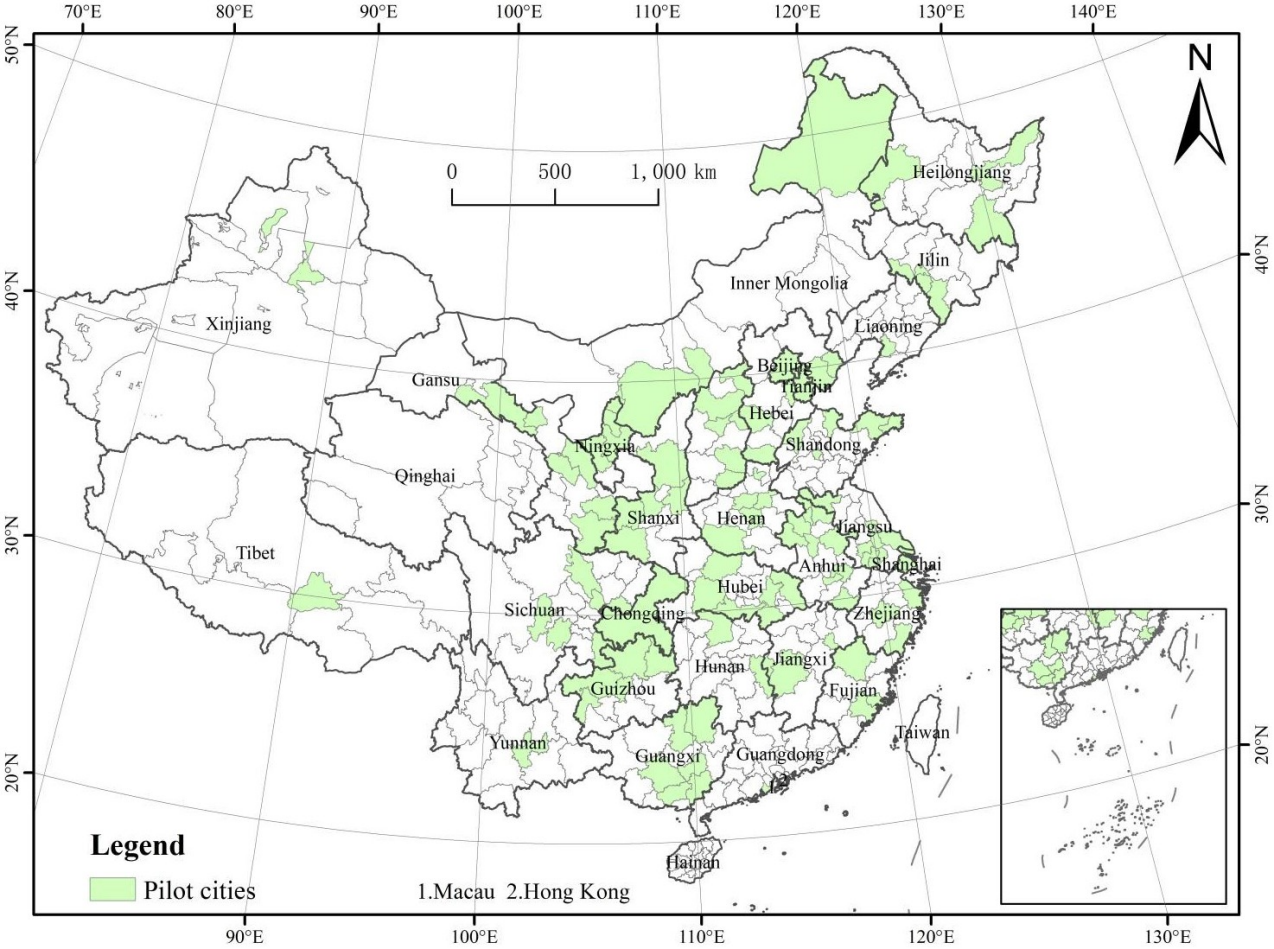
Pilot smart city construction in China. Map Audit Number: GS (2023) 2767 produced by Ministry of Natural Resources of the People’s Republic of China.

Smart city construction gradually entered the central government’s policy vision, and became an important milestone in China’s smart city construction. 2011, the Outline of the Twelfth Five-Year Plan for the National Economic and Social Development of the People’s Republic of China put forward the goal of "promoting the construction of digital cities, and improving the level of informatization and fine management and service". Under the guidance of the policy, in 2012, the State Council issued Several Opinions on Vigorously Promoting the Development of Informatization and Effectively Guaranteeing Information, making it clear that "it is necessary to promote the sharing of urban management information, promote the grid management model, accelerate the implementation of pilot demonstrations of smart grids, smart transportation and other pilot projects, and guide the development of the construction of smart cities". In November of the same year, China’s Ministry of Housing and Urban-Rural Development issued the "Notice on the Pilot Work of Smart Cities", identifying 90 cities such as Shanghai Pudong New Area and Beijing Dongcheng District as the first batch of national smart city pilots [44] At the same time, the Ministry of Urban-Rural Development issued the "Interim Management Measures for National Smart City Pilots" and the "National Smart City (District and Town) Pilot Indicator System (for Trial Implementation)", which enabled the construction of smart cities in China to move from the conceptual stage to practice. 2013 May, the second batch of pilots was expanded to 103 districts, and 2015 April, the third batch of pilots was further expanded to 84 districts. As illustrated in Fig 1, China’s smart city pilot projects have been implemented across the majority of provinces, reflecting the widespread implementation of China’s strategy to promote SCC. Moreover, the phased implementation of these projects provides a quasi-natural experiment setting. This enables the utilization of a difference-in-differences approach to investigate the health impacts of smart cities, capitalizing on variations in policy implementation across different regions and over time.

### 2.2 Data source and study population

The data come from the CHARLS database, and the regional socio-economic variables come from the China Statistical Yearbook, which can both be freely obtained from their official website.It is a large-scale interdisciplinary survey project hosted by the National Development Research Institute of Peking University and executed by the China Social Science Research Center. This initiative combines international aging survey methodologies with the specific requirements of China’s aging population, as well as the needs of public health, socio-economic fields, and other multidisciplinary areas when designing interviews and determining indicators. CHARLS employs a stringent multi-stage random sampling process. Initially, 150 districts and counties are chosen using the Probability Proportional to Size (PPS) method, taking into account factors such as region, urban-rural classification, and per capita income. Subsequently, three village-level units, totaling 450 units, are selected from each district or county. CHARLS-GIS facilitates the mapping and labeling of living units within buildings to establish a sampling frame for households. From each household, an individual aged 45 or older is randomly chosen as the primary respondent, and their spouse is automatically included in the sample. This approach is widely recognized as the predominant sampling methodology in developing countries. CHARLS has conducted baseline surveys across 28 provinces, autonomous regions, and municipalities directly under central government administration since 2011. Follow-up surveys were completed in 2013, 2015, and 2018, with subsequent follow-ups planned at 1–2 year intervals. By the end of the 2018 national tracking survey, the sample encompassed 19,000 respondents from 12,400 households. Given the robustness of the research, this study combines data from CHARLS for the years 2013, 2015, and 2018, aligned with the research objectives. In 2013, 10,629 households and 18,264 respondents were included with an 88.3% tracking rate; in 2015, there were 11,797 households and 20,284 respondents with an 85.8% response rate; and in 2018, 10,524 households and 17,970 respondents with an 86.46% response rate. (Refer to China Health and Aging Report (pku.edu.cn), P14). Since the CHARLS database started its survey in 2011 and the latest year is 2018, this paper sets the sample period as 2011–2018. At the same time, samples with serious missing values are deleted and continuous variables are indented by 1% up and down. After excluding samples under 60 years old, the final sample size was 3153.

### 2.3 Variables

#### 2.3.1 Health level of the elderly

This study drew on the methodology used in the study by Jie Pan and Xiaoyan Lei [45], the self-assessed health score(*Shealth*), as a health metric to explore the association between medical insurance and health, and it generated desirable findings. Another reason for choosing *Shealth* as the explanatory variable is that it serves as a comprehensive and valuable judgment of an individual’s current level of health. Therefore, this article will focus on the physical health of the elderly. Based on the results of individual choices in the survey question "What do you think of your health level?", the study assigned a value of 1 to those who choose "very bad", a value of 2 to those who choose "bad", a value of 3 to those who choose "fair", a value of 1 to those who choose "good" and a value of 5 to "very good", which is an ordered variable.

#### 2.3.2 Smart city pilots

On December 5, 2012, the Ministry of Housing and Urban-Rural Development of China officially announced the first batch of 90 smart city pilots. Following that, the second and third batches of pilot cities were successively unveiled in 2013 and 2015. Considering that the initial announcement occurred at the end of 2012, and the installation of information infrastructure and other construction activities for smart cities necessitates a certain amount of time, this paper establishes the timeline for both the first and second batches of city pilots as 2013. The core explanatory variable is the dummy variable of SCC. According to existing studies, *DID* is assigned a value of 1 if the SCC was piloted in year t in the elderly’s place of residence, and 0 otherwise [46].

### 2.4 Analytic approach

#### 2.4.1 Empirical model

Since SCC was piloted in batches and implemented gradually, it can be viewed as a quasi-natural experiment. In this study, a difference-in-variances model was used to identify the impact of SCC on the health of the elderly in the pilot policies at both time and area levels. The empirical model is constructed as follows.


$$Shealth_{\text{it}}=\alpha_{0}+\alpha_{1}DID_{it}+\alpha_{2}Control_{it}+\gamma_{i}+\mu_{t}+\varepsilon_{it}$$ (1)

In terms of intervention effectiveness evaluation, the DID model effectively combines "pre- and post- differences" and "whether there are differences", to some extent controlling the influence of certain factors other than intervention factors; At the same time, adding other covariates that may affect outcome variables to the model further controlled for certain "suspected" influencing factors in the intervention group and control group, to supplement the defect that "natural experiments" cannot be completely random in sample allocation [47]. Therefore, this article employed the DID model for empirical analysis to explore SCC’s impact on the health of the elderly.

In this model, *Shealth* was used to measure the health status of the elderly, with subscript i denoting the elderly, and t denoting the year. *DID* is a measure of SCC. If the location the elderly live piloted SCC system in year t, *DID* is assigned the value of 1, otherwise it is 0, and this variable is equivalent to the interaction term in the traditional double-difference method.

**Fig 2:**
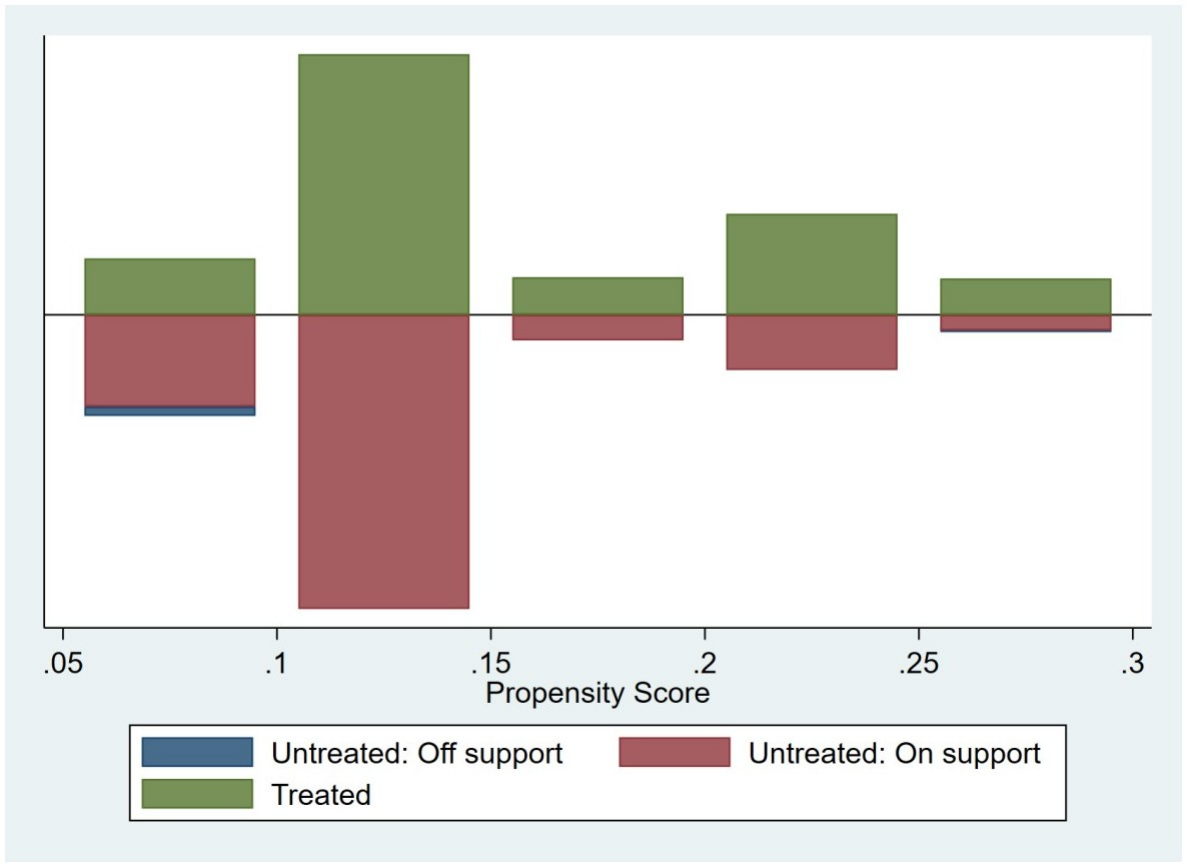
Propensity score distribution.

In addition, given that the selection of SCC pilots may be affected by the city’s economic status, population size, information technology development and other factors, this non-randomness of pilot selection may cause biased estimation results. Therefore, the study utilized propensity score matching (PSM) and subsequently employs the PSM-DID method to assess the pilot effect of smart cities more accurately. To test the applicability of the PSM-DID method for policy assessment, the article employs nearest-neighbour matching (1:1) for matching and utilizes the Logit model to estimate the propensity score value of each city (the distribution of the propensity score value is depicted in Fig 2). Importantly, the probability distribution between the treatment group and the control group remains consistent after matching, indicating the effectiveness of PSM. After discarding the unmatched samples, a total of 3100 samples are obtained, with 419 samples in the experimental group and 2681 samples in the control group.

#### 2.4.2 Control variables

The selection of control variables was grounded on the study by Wang Yizhen [48]which identified their value in comparable contexts, *Control* represents the set of control variables, including a series of individual characteristics of the elderly and regional macro-socioeconomic variables. Individual characteristics variables of the elderly include age(*age*), gender(*gender*), marital status(*married*), whether they registered in urban area(*hukou*), whether they smoke(*smoke*), and whether they drink(*drink*), whether they have a chronic illness(*mxb*). On this basis, macroeconomic and social variables were added, including the economic growth rate (*rgdp*), financial investment intensity(*fiscal*), population density(*pop*)and level of industrial structure(*cyjp*). Among them, the economic growth rate, the financial investment intensity, and the level of industrial structure can affect the income and services received by the elderly, thereby influencing their health [49,50], In contrast, population density is negatively correlated with elderly health [51]. This paper controlled for the regional fixed effects *γ*_i_ and year fixed effects *μ*_t_. The coefficient *α*_1_ measures the effect of the implementation of the SCC, reflecting the net effect of the impact of the SCC on the health of the elderly. The descriptive statistics of the data are shown in Table 1.

**Table 1:** Descriptive statistics for each of the main variables.

Variable	Obs	Mean	Std. Dev.	Min	Max
Shealth	3100	3.163	0.883	1	5
did	3100	0.135	0.342	0	1
age	3100	68.25	6.462	60	97
gender	3100	0.451	0.498	0	1
edu	3100	2.294	1.196	1	5
married	3100	0.219	0.414	0	1
drink	3100	0.305	0.46	0	1
smoke	3100	0.227	0.419	0	1
hukou	3100	0.774	0.418	0	1
mxb	3100	0.87	0.336	0	1
rgdp	3100	9.073	1.987	5.54	17.88
fiscal	3100	4.615	1.683	1.512	10.295
pop	3100	466.816	238.198	56.6	1055.84
cyjg	3100	0.428	0.054	0.348	0.536

## 3. Results

### 3.1 Baseline results

This paper examined the impact of SCC on the health of the elderly. The regression results of the model are presented in Table 2. In Table 2, column (1) gives the estimation results with control variables, both individual characteristics variables of the elderly and macroeconomic and social variables. The former included age(*age*), gender(*gender*), educational attainment(*edu*), marital status(*married*), whether they drink(*drink*), whether they smoke(*smoke*), whether they registered in urban areas(*hukou*) and whether they have a chronic illness(*mxb*). The latter include the economic growth rate (*rgdp*) financial investment efforts(fiscal), population density per square kilometer(pop) and the share of the secondary industry (*cyjp*). The coefficient of *DID* is significantly positive at the 5% level, indicating that SCC improves the health of the elderly.

**Table 2:** Baseline regression results.

	(1)
	Shealth
did	0.096**
	(0.046)
age	-0.002
	(0.002)
gender	-0.003
	(0.04)
edu	0.01
	(0.015)
married	0.069*
	(0.039)
drink	-0.111***
	(0.035)
smoke	-0.027
	(0.044)
hukou	0.243***
	(0.04)
mxb	0.178***
	(0.009)
rgdp	0a.009
	(0.011)
fiscal	-0.038***
	(0.012)
pop	0***
	(0)
cyjg	0.555
	(0.581)
_cons	2.731***
	(0.374)
Observations	3100
R-squared	0.146

Note:

***, **, and * denote significant at the 1%, 5%, and 10% levels respectively, and the numbers in parentheses are robust standard errors clustered by firms, the same below.

### 3.2 Robustness checks

To test the robustness of the previous empirical results, the study performed the following robustness tests.

#### 3.2.1 Parallel trend test

The study leveraged the work of Ferrara [52] to assess the validity of the assumption using a "counterfactual" approach that manipulated the timing of policy implementation. Due to constraints in data availability, the SCC schedule was synchronized across all districts one year in advance. If the core variable DID ceases to be statistically significant, it implies that the experimental and control groups exhibited a common trend before the implementation of the check. The results of the "counterfactual" regression in Column (1) of Table 3 detail that the core explanatory variable didpre1 was not statistically significant one year prior to SCC implementation, This means that before the policy implementation, the experimental group and the control group satisfied the parallel trend assumption, indicating that the parallel trend test was successful.

**Table 3:** Parallel trend test.

	(1)
	Shealth
didpre1	-0.031
	(0.16)
age	-0.001
	(0.002)
gender	0.002
	(0.041)
edu	0.009
	(0.015)
married	0.056
	(0.039)
drink	-0.123***
	(0.035)
smoke	-0.033
	(0.044)
hukou	0.205***
	(0.045)
mxb	0.176***
	(0.009)
rgdp	-0.009
	(0.015)
fiscal	-0.026
	(0.024)
pop	-0.001
	(0.001)
cyjg	1.081
	(1.759)
_cons	2.782***
	(0.987)
Observations	3100
R-squared	0.16

#### 3.2.2 Placebo test for exclusion of randomized outcomes

**Fig 3:**
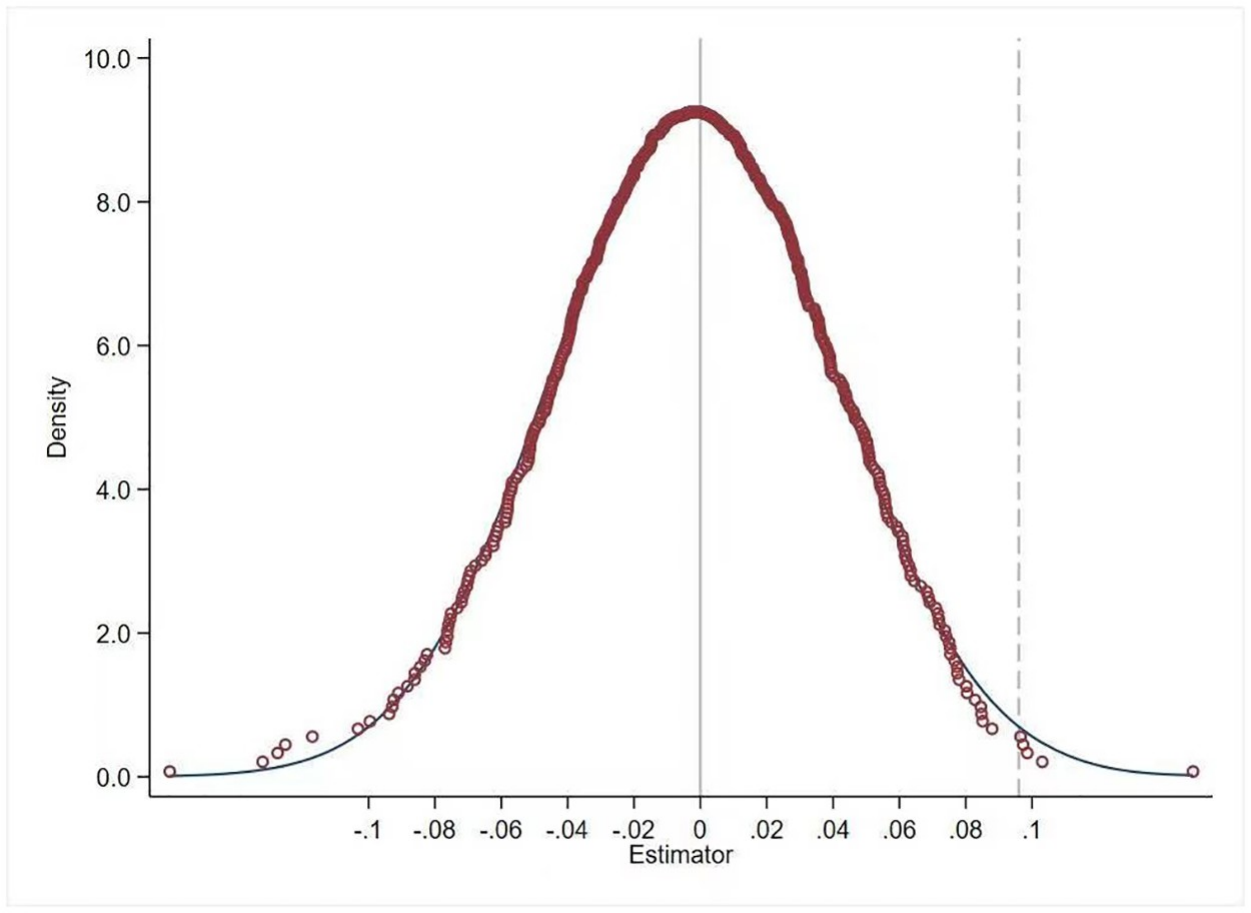
Placebo test chart.

To rule out the effect of randomized results, drawing on Chetty [53] approach, the year and region in which SCC was implemented were randomized, and this process was iterated 500 times for the placebo test. The results, as shown in Fig 3, manifest that the randomized simulation yields a distribution of regression coefficients centered around 0, while the coefficients of the benchmark regression are entirely independent of this coefficient distribution. This represents that the empirical results of this paper are not due to randomness or chance occurrences.

### 3.3 Heterogeneity tests

#### 3.3.1 Living with their children and not living with their children

Living with children is a serious factor affecting the health of the elderly. Elderly people who live with their young children could have a happier, easier and safer life. In this study, health problems were detected and treated in a timely manner, and risks such as broken bones were avoided. It was found that SCC might have a more prominent health impact on the elderly without being accompanied by children. Specifically, columns (1) and (2) of Table 4 show the empirical results of regressing the samples of the elderly whether living with their children or not. In detail, column (1) manifests the test results of the elderly living with their children, with a coefficient of 0.128, insignificant at the 10% confidence level, while column (2) reveals the results of the elderly not living with their children, with a coefficient of 0.098, significant at the 10% confidence level. In summary, this means that the SCC has a greater impact on the health of the elderly not living with their children.

**Table 4:** Heterogeneity between living with their children and not living with their children.

	(1)	(2)
	Living with children	Not living with children
	shealth	shealth
did	0.128	0.098*
	(0.081)	(0.056)
age	0.001	-0.003
	(0.004)	(0.003)
gender	-0.104	0.05
	(0.068)	(0.05)
edu	0.003	0.013
	(0.024)	(0.018)
married	0.131**	-0.004
	(0.059)	(0.052)
drink	-0.046	-0.144***
	(0.06)	(0.043)
smoke	0.119	-0.099*
	(0.074)	(0.055)
hukou	0.275***	0.212***
	(0.067)	(0.051)
mxb	0.177***	0.179***
	(0.015)	(0.011)
rgdp	0.007	0.003
	(0.016)	(0.015)
fiscal	-0.025	-0.051***
	(0.02)	(0.015)
pop	0***	0
	(0)	(0)
cyjg	1.032	0.294
	(0.925)	(0.766)
_cons	2.16***	3.352***
	(0.586)	(0.5)
Observations	1209	1891
R-squared	0.152	0.158

#### 3.3.2 Rural hukou and urban hukou

Given the current household registration system and prevailing economic and social conditions, the mobility of elderly individuals was limited. This was primarily observed in the fact that a majority of elderly individuals with rural household registration reside in rural areas. In this case, the stark differences in urban and rural infrastructure created distinct living environments for the elderly in cities and rural areas. Regarding the health status of the elderly, those dwelling in urban areas had less space to maneuver, and the air quality was poorer—factors inconducive to the amelioration of the physical health of the elderly. Consequently, there existed potential heterogeneity in the impact of SCC on rural and urban denizens. Columns (1) and (2) in Table 5 demonstrate the empirical results of regressing the samples of the elderly people with rural hukou or urban hukou respectively. Besides, column (1) shows the regression results of elderly people with rural hukou, whose coefficient is insignificant at 10% confidence level, while column (2) showcases the regression results of elderly people with urban hukou, with a coefficient of 0.105, significant at 10% confidence level. In summary, this means that the impacts of SCC on the health of urban elderly people are more prominent.

**Table 5:** Heterogeneity between rural and urban.

	(1)	(2)
	Rural hukou	Urban hukou
	shealth	shealth
did	0.045	0.105*
	(0.079)	(0.06)
age	-0.002	-0.001
	(0.005)	(0.003)
gender	-0.073	0.021
	(0.08)	(0.047)
edu	0.011	0.004
	(0.026)	(0.018)
married	-0.065	0.104**
	(0.079)	(0.044)
drink	-0.182**	-0.091**
	(0.073)	(0.04)
smoke	0.053	-0.055
	(0.089)	(0.051)
mxb	0.167***	0.18***
	(0.017)	(0.011)
rgdp	0.055**	-0.002
	(0.026)	(0.012)
fiscal	-0.059**	-0.03**
	(0.026)	(0.014)
pop	0	0***
	(0)	(0)
cyjg	1.846	.135
	(1.526)	(0.665)
_cons	1.761*	3.477***
	(0.899)	(0.928)
Observations	701	2399
-squared	0.178	0.132

### 3.4 Mechanism tests

In Section 2.1, this paper conducted a theoretical analysis of the impact mechanism of smart cities on elderly health. Subsequently, the study empirically examined the influence of smart cities on the construction of urban leisure infrastructure, the level of medical service provision, urban environmental protection and information and communication technology infrastructure development.

#### 3.4.1 Analytical framework

**Fig 4:**
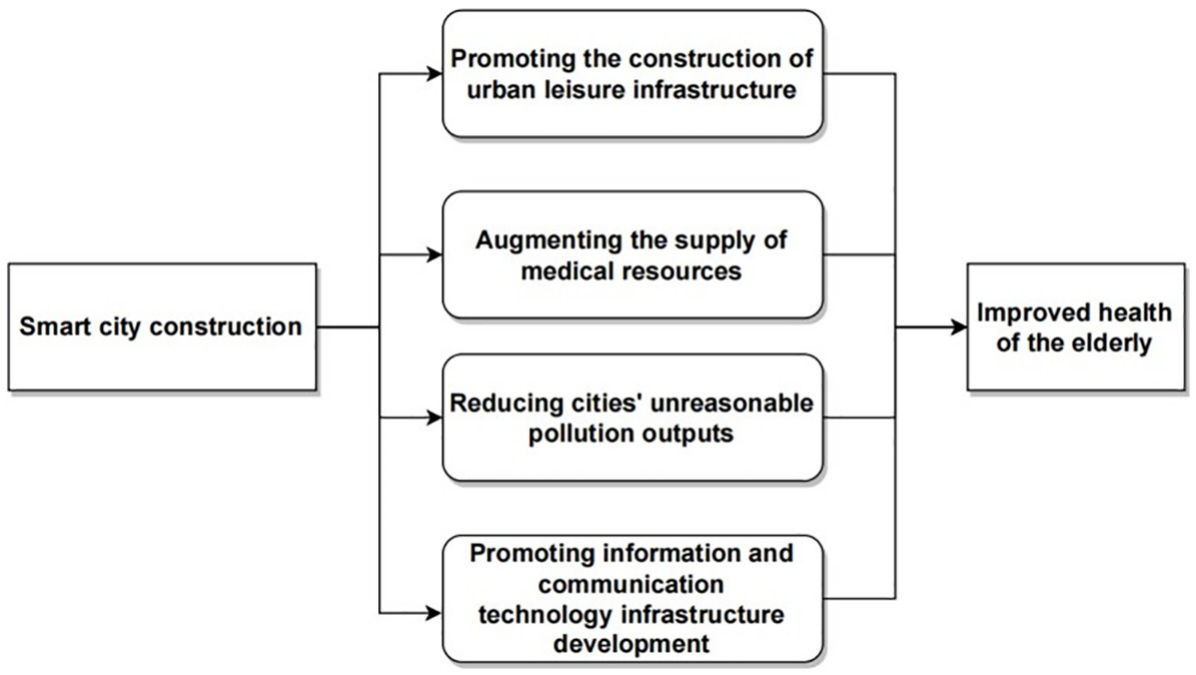
The impact mechanism of SCC on elderly health is illustrated.

The objective of this paper is to investigate the impact of smart cities on the health of the elderly, recognizing that the influence pathways of smart cities on elderly health are multidimensional. Based on this perspective, and drawing insights from policies and existing literature, we aim to preliminarily explore the theoretical framework through which smart cities affect the health of the elderly. The Chinese government, in its 2014 "Guiding Opinions on Promoting the Healthy Development of Smart Cities," highlighted that SCC should prioritize efforts in infrastructure, public services, urban governance, and network development. Giffinger was an early proponent who delineated the constitutive elements from a goal-oriented perspective, incorporating aspects such as smart economy, smart living, smart people, smart environment, smart mobility, and smart governance into the framework of SCC [54]. Similarly, Bolívar and Meijer proposed that the goals of smart cities are reflected in economic growth, citizen-centric service provision, and the enhancement of citizen interaction [55]. Nicolas further refined this perspective by quantifying the dynamic impact of key factors promoting smart cities on performance goals and identified urban efficiency, environmental sustainability, livability, and economic competitiveness as the primary development goals of SCC [56]. Thus, it is clear that SCC, viewed from both policy content and urban development goals, is intricately linked to factors influencing the health of the elderly. Significantly, existing research highlights that the construction of urban leisure infrastructure [57], the level of medical service provision [58], urban environmental protection [59] and information and communication technology infrastructure [60,61] in cities all play roles in influencing the elderly’s health status. Drawing from this, the paper will analyze the impact of smart cities on the health of the elderly across four dimensions: the construction of urban leisure infrastructure, the level of medical service provision, urban environmental protection and the development of information and communication technology infrastructure. The impact mechanism of SCC on elderly health is illustrated in Fig 4.

#### 3.4.2 The mediating mechanism test of the construction of urban leisure infrastructure

Drawing on existing research, this paper employed per capita urban green space area as a proxy variable for the level of urban leisure infrastructure construction [62,63]. Table 6 presents the regression results for the impact of smart cities on the level of leisure infrastructure construction. The *DID* coefficient is 0.762, significant at the 5% level, indicating that smart cities have increased per capita urban green space area, thereby promoting the construction of urban leisure infrastructure.

**Table 6:** Regression results of the impact of the SCC on the construction of urban leisure infrastructure.

	(1)
	Green space per person
did	0.762**
	(0.373)
rgdp	0.006
	(0.056)
fiscal	0.106
	(0.101)
pop	-0.002***
	(0.001)
cyjg	0.031*
	(0.018)
_cons	10.408***
	(3.021)
Observations	945
R-squared	0.304

#### 3.4.3 The mediating mechanism test of the level of medical service provision

The backbone of China’s healthcare system often relies on administrative hierarchies, resulting in medical and healthcare institutions in cities of the same administrative level showing little difference in quantity. Nevertheless, there exists a considerable gap in hospital scale, with notable disparities in bed and physician numbers. Therefore, this paper assessed the level of medical service provision by employing the number of healthcare technical personnel and beds per thousand people [64]. Table 7 displays the regression outcomes. In the initial column, the analysis assesses the influence of smart cities on the number of healthcare technical personnel per thousand individuals, revealing a *DID* coefficient of 0.275 significantly significant at the 1% level. This implies that smart cities have increased the number of healthcare technical personnel per thousand people. In the second column, the impact of smart cities on the number of healthcare technical personnel per thousand people is assessed, revealing a *DID* coefficient of 0.099 significant at the 10% level. This indicates that smart cities have significantly elevated the number of healthcare medical beds per thousand people.

**Table 7:** Regression results of the impact of the SCC on supply of medical resources.

	(1)	(2)
	mbed	mmpop
did	0.275***	0.099*
	(0.091)	(0.054)
rgdp	-0.051***	-0.045***
	(0.012)	(0.006)
fiscal	0.03	0.031**
	(0.023)	(0.014)
pop	-0.001***	0***
	(0)	(0)
cyjg	0.009**	-0.002
	(0.004)	(0.002)
_cons	-188.099***	2.674***
	(41.769)	(0.115)
Observations	954	954
R-squared	0.187	0.121

#### 3.4.4 The mediating mechanism test of urban environmental protection

Given the availability of regional and municipal emissions data, the study selects wastewater discharge and exhaust emission in each prefecture-level city as mediating variables to explore the impact of the SCC on the above variables. The regression results, shown in Table 8, indicate that the SCC significantly reduces industrial wastewater emissions and exhaust emissions, which reveals that the intensity of regional emissions may be a possible pathway through which SCC influences the health of the elderly. In detail, in the first column, the influence of smart cities on wastewater discharge is significant at the 5% level with a *DID* coefficient of -0.159. Besides, according to the second column, the effect of smart cities on exhaust emission is also significant at the 5% level, yielding a *DID* coefficient of -0.375. This indicates that smart cities have significantly suppressed urban pollution emissions and protected the urban environment.

**Table 8:** Regression results of the impact of the SCC on environmental protection.

	Wastewater emissions	Exhaust emissions
did	-0.159**	-0.4472***
	(0.074)	(0.1559)
rgdp	-0.005	-0.1164***
	(0.008)	(0.0166)
fiscal	0.092***	0.2698***
	(0.018)	(0.0382)
pop	0.001***	0.0006***
	(0)	(0.0002)
cyjg	0.02***	0.0359***
	(0.003)	(0.0063)
_cons	6.304***	11.1169***
	(0.158)	(0.3068)
Observations	954	954
R-squared	0.266	0.189

#### 3.4.5 The mediating mechanism test of information and communication technology infrastructure development

Existing studies often select the number of Internet broadband access users and the number of cell phone users at the end of the year to measure the level of information and communication technology development [65,66], but in 2015, data from China’s Ministry of Industry and Information Technology (MIIT) showed that the number of China’s cell phone users reached 1.306 billion in that year, and the penetration rate of cell phone users reached 95.5 per 100 people, an increase of 1 per 100 people compared with 2014. Therefore, this paper selected the number of Internet broadband access users to measure the level of information and communication technology construction.

The regression results are shown in the Table 9, and the coefficients are significant at the 1% level for evidence and positive, indicating that Internet penetration increases with the construction of smart cities.

**Table 9:** Regression results of the impact of the SCC on internet access.

	(1)
	Internet access
did	16.075***
	(4.333)
rgdp	-7.206***
	(0.491)
fiscal	7.355***
	(1.122)
pop	0.017***
	(0.005)
cyjg	-0.695***
	(0.185)
_cons	211.782***
	(9.179)
Observations	954
R-squared	0.292

## 4. Discussion

This study signified the inaugural assessment utilizing a multi-period DID model to examine the correlation between SCC and the health status of the elderly. Leveraging CHARLS statistical data from 2011 to 2018, our study explored the association between SCC and elderly health. Employing a multi-period DID model, complemented by parallel trends and placebo tests to mitigate endogeneity concerns between explanatory and dependent variables, this study proved the significant positive impact of SCC on the health levels of the elderly. Our results in this study are similar to those of Wang et al., which suggest that urban environmental governance is beneficial for improving the health of the elderly [67]. Differently, this article is an extension of Wang et al.’s research. Wang et al. only investigated the health effects of low-carbon urban environmental governance, while this article not only explores the impact of SCC on environmental governance, but also explores other impact effects. Furthermore, this study explored the impact mechanisms through which smart city development influences the health levels of the elderly. The findings indicate that SCC facilitates the construction of urban leisure infrastructure (such as per capita parks and green space areas), enhances urban medical service provision (such as the number of beds and medical personnel per thousand people), promotes urban environmental protection (by restraining industrial waste and sulfur dioxide emissions and improving air quality), and fosters the development of urban information and communication technology infrastructure. SCC may improve the health levels of the elderly by influencing the factors mentioned above. The mechanism study in this paper not only clarified the pathways through which SCC affect the health of the elderly but also expanded the research on the impact of SCC. Current research extensively investigates the positive impacts of smart city development on smart healthcare [68,69], support for the disabled [70,71], and urban air quality [72]. These studies predominantly focus on the application of smart city frameworks in enhancing public health at a practical application level. However, there is a notable absence of empirical research exploring the direct health effects of smart cities. In this paper, we leverage a sample from China to examine the health outcomes associated with smart cities. This approach is relatively novel, as there has been limited exploration using population data from other countries or regions to study the health implications of smart city initiatives. This aspect represents a unique contribution of our study to the existing body of literature.

In the section on heterogeneity analysis, we found that SCC is more conducive to improving the physical health of the elderly not living with their children. Several plausible explanations may account for this finding. Firstly, the elderly without direct care from their children face many obstacles to survival challenges [73,74], and smart cities can provide improved transportation, delivery and shopping services. This, in turn, reduces the daily life pressures on the elderly, making it easier for them to acquire essential items like food, medication, and other necessities. Secondly, the supportive role of smart devices in the lives of the elderly living alone has long been proven [75,76]. Smart cities can provide a variety of smart assistive devices, such as smart hearing aids, smart glasses and smart electric wheelchairs. These devices aid elderly individuals in coping with the challenges of declining physical functions, enhancing their independence and quality of life. Lastly, remote monitoring systems for collecting information about the lives of solitary elderly individuals have been widely used in the field of elderly health [77,78]. The health monitoring technologies embedded in SCC, encompassing health sensors, smart medical devices and remote medical services, can help the elderly better manage their health conditions. They can monitor vital signs at any time and receive timely medical advice and interventions.

Moreover, the results suggest that the health promotion effects of SCC are more pronounced among elderly individuals with urban household registrations as compared to those with rural household registrations. One possible reason is that, before SCC, the urban elderly, in contrast to their rural counterparts or those on the outskirts of cities, were exposed to higher levels of air pollution caused by significant vehicular movement and industrial activities [79,80] as indicated in the mechanism study that smart cities curb polluting emissions, which will benefit the health of the urban elderly. In addition, smart cities rely on technological infrastructures such as advanced information and communications technology, Internet of Things devices, big data analytics, etc., which are usually more developed and widespread in urban areas. Urban seniors are thus more likely to benefit directly from smart city conveniences, such as telemedicine services, smart homes, and online social networking platforms, which can improve their quality of life and convenience. In contrast, rural areas may be less likely to reap the direct benefits of these technologies due to inadequate infrastructure. Moreover, appropriate physical activities are beneficial for the health of the elderly [81]. Before smart cities were built, suboptimal urban planning implied that the urban elderly had limited outdoor spaces for exercise. SCC, with its capacity to optimize city spatial layouts, can create more exercise facilities as well as parks, which will attract more older people to participate in outdoor exercises and improve their health.

In terms of the outcome of the mechanism test. Firstly, this paper finds that SCC promotes the construction of urban leisure infrastructure. Meanwhile, existing studies show that, well-designed parks and green spaces offer a conducive environment for residents’ physical exercise [82]. Besides, moderate outdoor activities are beneficial for the health of the elderly both psychologically [83,84] and physically [85,86]. Hence, SCC can improve the health of the elderly by promoting the development of urban leisure infrastructure. Furthermore, empirical studies have shown that smart city construction increases the supply of healthcare resources. This is supported by practice, where SCC emphasises the transformation of community public service information systems, the establishment of information service systems for home care, elderly care, community nursing, patient care, and so on, and the use of technologies such as big data analytics and artificial intelligence to more accurately predict healthcare demand and optimise the allocation of healthcare resources [87]. A wealth of research indicates that augmenting the supply of medical resources is significant in promoting health [58,88]. Therefore, SCC may have improved the health of the elderly by enhancing the availability of healthcare resources for them. Additionally, this paper manifested that SCC reduces urban emissions. This is attributed to the deployment of numerous environmental monitoring sensors in smart cities. Specifically, by deploying sensors to gather real-time data on air quality indicators such as PM2.5, PM10, nitrogen dioxide, sulfur dioxide, among others, smart cities can automatically detect and report environmental violations, thereby enhancing the efficiency and effectiveness of environmental protection enforcement [89], This ultimately helps the reduction in pollutant emissions, which is crucial for maintaining a healthy environment for the elderly. Air pollutants, particularly particulate matter and ozone, can directly irritate the respiratory tract, leading to various respiratory diseases such as chronic obstructive pulmonary disease (COPD) [90], asthma [91], and pneumonia [92]. Furthermore, air pollution may be associated with the development of conditions like dementia [93] and other neurodegenerative diseases [94]. Thus, SCC may have improved the health of older people by reducing the scale of urban pollution and improving air quality. Finally, the paper finds that SCC promotes information and communication infrastructure development. Specifically, information and communication technology facilitates the feasibility of remote healthcare, allowing elderly individuals to access medical consultations and treatment from their homes. This assumes heightened significance for individuals facing mobility challenges or residing in geographically isolated regions. Notably, healthcare professionals can remotely monitor essential physiological parameters, such as heart rate, blood pressure, and blood glucose levels, providing a thorough assessment of the elderly person’s health [95]. Moreover, information and communication technology empowers the elderly with increased knowledge about disease prevention, care, and coping strategies [96], positively influencing their health. Additionally, it supports enhanced social connectivity [97] and reduced social isolation among the elderly [98], contributing to improved mental well-being. Thus, SCC strategically promotes information and communication technology infrastructure development, presenting a comprehensive and integrated strategy to improve health outcomes for the elderly.

## 5. Limitations

The study acknowledged certain limitations that warrant attention. First, despite controlling for nine variables encompassing the demographic characteristics of the elderly and features of regional economic development, these variables may not comprehensively cover all fundamental confounding factors influencing elderly health. Unobservable control variables may increase selection bias and affect the regression results. Second, our study’s data covers the years 2011 to 2018, without exploring the impact of SCC on geriatric health during the covid-19 epidemic. Future research endeavors are poised to rectify this limitation by assimilating survey data from the CHARLS database collected during the pandemic. Finally, limited to the availability of data, this paper only analyzes the impact of SCC on the supply level of healthcare resources, leaving unexplored the developmental trajectory of smart healthcare—a dimension not accommodated within our existing analytical framework probing causal mechanisms.

## 6. Conclusion and policy implication

The results show that smart city construction enhances the health of the elderly. Specifically, the construction achieved a significant improvement in the physical health of the elderly who did not live with their children. The health promotion effect of the smart city was more significant for the urban elderly than for the rural elderly. The elucidated mechanisms of influence suggest that smart cities bring about their effects through the promotion of urban leisure infrastructure, enhancement of medical service provision, advancement in urban environmental protection and stimulation of urban information and communication technology infrastructure development.

Based on the above research conclusions, this article draws the following insights: First, the provision of medical care in smart cities needs to be enhanced. Smart cities have the capability to gather extensive medical and health data from elderly individuals through various means, including medical records, health monitoring devices, and healthcare service utilization information. By employing advanced big data analysis techniques, it can be thoroughly analyzed to identify the medical needs of different elderly demographics, predict potential health risks, and tailor medical provisions accordingly; Secondly, advanced information technology should be harnessed to elevate telemedicine services to a more sophisticated level. Smart cities can offer telemedicine consultations and diagnostics through internet platforms and mobile communication technologies. This is especially importatnt for elderly individuals with limited mobility, residing in remote rural areas, and lacking familial support, enabling them to engage in timely consultations with healthcare professionals via video calls and health monitoring devices. In cases of health emergencies such as falls or heart attacks, immediate notifications can be automatically relayed to emergency service centers and family members through smart wearable devices or home emergency buttons; Lastly, there is a pressing need to enhance the efficiency and effectiveness of urban environmental management. Through the deployment of sensor networks for environmental monitoring, critical indicators such as air quality, water quality, and noise levels can be monitored in real-time, ensuring prompt detection and resolution of environmental issues. By implementing smart waste collection and sorting technologies, such as sensor-equipped waste bins, waste accumulation levels can be monitored effectively, waste collection processes can be optimized, resource recovery rates can be increased, and the environmental impact of waste disposal can be minimized. Ultimately, these efforts contribute to the creation of a more sustainable and livable urban environment, beneficial for promoting healthy aging among the elderly.

## 7. Future research directions

Future research directions may include the following areas: Firstly, an in-depth analysis of the differential impacts of smart cities on the health of various population groups should be pursued. This research could be expanded to include populations in other countries and regions, such as adolescents and the working adult population, to analyze the effects of smart cities on their health and to explore potential urban-rural disparities; Secondly, the cost-effectiveness of smart city construction should be evaluated, particularly in terms of its economic benefits in improving the health of the elderly. This includes cost savings (e.g., reduced healthcare expenditures) and enhancements in the quality of life, thereby providing investment decision support for urban planners and policymakers; Thirdly, case studies and comparative analyses should be conducted. Through case studies, the implementation process, challenges faced, and success stories of specific smart city projects can be examined in detail. Cross-national comparative studies could analyze the strategies and outcomes of smart city developments in different countries and regions in promoting the health of the elderly, extracting successful elements and experiences for a more comprehensive understanding of the impact of smart cities on elderly health; Lastly, the long-term impacts and sustainability assessments of smart cities on elderly health should be tracked, including continuous improvement in mental health and enhancements in the quality of life. This would provide a deeper understanding of the enduring effects of smart city initiatives on public health.

## Supporting information

S1 Dataset (DTA)

## References

[1] Li HZ, Yang X, Zhu Y. The general research report of chinese strategic for dealing with population aging. Science Research Aging. 2015;3:4–35.

[2] Du P, Li L. Long-term trends projection of China’s population aging in the New Era. Journal of the Renmin University of China. 2021;35:96–109.

[3] Eibich P. Understanding the effect of retirement on health: Mechanisms and heterogeneity. Journal of Health Economics. 2015;43:1–12. doi: 10.1016/j.jhealeco.2015.05.001 26079117

[4] Merson MH, Black RE, Mills AJ. Global health: Diseases, programs, systems, and policies. Jones Bartlett Publishers. 2011.

[5] Xie E. Income-related health and medical service utilization inequality study. Economic Research. 2009;44:92–105.

[6] Wilkinson RG. Socioeconomic determinants of health: Health inequalities: relative or absolute material standards? British Medical Journal. 1997;314:591–595. 9055723 10.1136/bmj.314.7080.591 PMC2126067

[7] Qi LS. Income inequality and health: the impact of urban-rural disparities and occupational status. Economic Research. 2006:16–26.

[8] Young GJ, Cohen BB. Inequities in Hospital Care, the Massachusetts Experience. Inquiry. 1991:255–262. 1833336

[9] Wang XJ, Zheng C. The impact of health insurance on medical expenditure and health among the elderly. Economic Research. 2014;40:65–75.

[10] Zhang Y, Wang XJ. Study on health influencing factors and insurance mechanisms of urban elderly——Based on the analysis of CLHLS survey data. Exploration of Financial Theory. 2019:71–80.

[11] Wang FQ. Socioeconomic status, lifestyle and health inequality. Society. 2012;32:125–143.

[12] Zhang C, Zhang D. The impact of social activity participation on their health——based on CHARLS 2011 year data. Population and Economy. 2016:55–63.

[13] Yu YY, Feng J. Impact of family care on medical service utilization in the elderly. Economics. 2018;17:923–948.

[14] Du BF, Wang X. Evolution, regional differences and influencing factors analysis of health inequality among the elderly. Population Studies. 2013;37:81–90.

[15] Sun HB, Zhao X. Effect of living conditions on the health of urban older adults. Journal of Dalian University of Technology. 2018;39:121–128.

[16] Yang XS, Liu HL. Impact of geographic allocation of healthcare resources on health status: an empirical analysis based on provincial panel data. China’s Health Economy. 2016;35:63–65.

[17] Stevenson M, Thompson J, de Sá TH, Ewing R, Mohan D, McClure R, et al. Land use, transport, and population health: estimating the health benefits of compact cities. The Lancet. 2016, 388, 2925–2935. 10.1016/S0140-6736(16)30067-8 PMC5349496 27671671

[18] World Health Organization. The First Ten Years Of The World Health Organization. Geneva: World Health Organization. 1958.

[19] Lytle LA, Sokol RL. Measures of the food environment: A systematic review of the field, 2007–2015. Health Place. 2017;44:18–34. doi: 10.1016/j.healthplace.2016.12.007 28135633

[20] Barton H, Grant M, Guise R. Shaping Neighbourhoods: For Local Health And Global Sustainability. London: Spon Press. 2013.

[21] De Vries S, Van Dillen SM, Groenewegen PP, Spreeuwenberg P. Streetscape greenery and health: Stress, social cohesion and physical activity as mediators. Social Science & Medicine. 2013;94:26–33. doi: 10.1016/j.socscimed.2013.06.030 23931942

[22] Grigsby-Toussaint DS, Chi SH, Fiese BH. Where they live, how they play: Neighborhood greenness and outdoor physical activity among preschoolers. International Journal of Health Geographics. 2011;10:1–10. 22165919 10.1186/1476-072X-10-66 PMC3278349

[23] Mooney SJ, Joshi S, Cerdá M, Kennedy GJ, Beard JR, Rundle AG. Neighborhood disorder and physical activity among older adults: A longitudinal study. Journal of Urban Health 2017;94:30–42. doi: 10.1007/s11524-016-0125-y 28108872 PMC5359178

[24] Ewing R, Schmid T, Killingsworth R, Zlot A, Raudenbush S. Relationship between urban sprawl and physical activity, obesity, and morbidity. Health Place. 2014;26:118–126. 24434082 10.1016/j.healthplace.2013.12.008

[25] Frank LD, Sallis JF, Conway TL, Chapman JE, Saelens BE, Bachman W. Many pathways from land use to health: Associations between neighborhood walkability and active transportation, body mass index, and air quality. Journal of the American Planning Association. 2006;72:75–87.

[26] Zimring C, Joseph A, Nicoll GL, Tsepas S. Influences of building design and site design on physical activity: research and intervention opportunities. American Journal of Preventive Medicine. 2005;28:186–193. doi: 10.1016/j.amepre.2004.10.025 15694527

[27] Lake A, Townshend T. Obesogenic environments: exploring the built and food environments. Journal of the Royal Society for the Promotion of Health. 2006;126:262–267. doi: 10.1177/1466424006070487 17152319

[28] Tian L, Su SL, et al. Urban and rural planning and public health. Beijing: China State Engineering and Construction Press. 2019.

[29] Yang LS, Li HR, Li YH, et al. Major areas and advances in research on medical geography and environmental health. Progress in Geographical Science. 2010;29:31–44.

[30] Kwate NOA, Yau CY, Loh JM, Williams D. Inequality in obesogenic environments: Fast food density in New York City. Health Place. 2009;15:364–373. 18722151 10.1016/j.healthplace.2008.07.003

[31] Sass V, Kravitz-Wirtz N, Karceski SM, Hajat A, Crowder K, Takeuchi D. The effects of air pollution on individual psychological distress. Health Place. 2017;48:72–79. doi: 10.1016/j.healthplace.2017.09.006 28987650 PMC6023621

[32] Buoli M, Grassi S, Caldiroli A, Carnevali GS, Mucci F, Iodice S, et al. Is there a link between air pollution and mental disorders? Environment International. 2018;118:154–168. doi: 10.1016/j.envint.2018.05.044 29883762

[33] Min JY, Kim HJ, Min KB. Long-term exposure to air pollution and the risk of suicide death: A population-based cohort study. Science of the Total Environment. 2018;628:573–579. doi: 10.1016/j.scitotenv.2018.02.011 29454198

[34] Li CJ, Ma J, Cai YW, Guan MB. The impact of residential environment and noise pollution on residents’ mental health——Take Beijing as an example. Advances in Geographical Science. 2019;38:1103–1110.

[35] O’Donnell O, van Doorslaer E, Wagstaff W, Lindelow M. Analyzing Health Equity Using Household Survey Data: A Guide to Techniques and Their lmplementation. Washington, DC: The World Bank Google Scholar 2008.

[36] Onda K, Lo Buglio J, Bartram J. Global Access to Safe Water: Accounting for Water Quality and The Resulting lmpact on MDG Progress. International Journal of Environment Research and Public Health. 2012;9:880–894. 10.3390/ijerph9030880 PMC3367284 22690170

[37] Frumkin H, Frank LD, Jackson RJ. Urban sprawl and public health: Designing, planning, and building for healthy communities. Island Press. 2004.

[38] Ramirez LKB, Hoehner CM, Brownson RC, Cook R, Orleans CT, Hollander M, et al. lndicators of activity-friendly communities: an evidence-based consensus process. American Journal of Preventive Medicine. 2006;31:515–524. 17169714 10.1016/j.amepre.2006.07.026

[39] Zhao J, Wang L, Guo K. Impact of smart health systems on the behavior of older adults under community healthcare. Front. Public Health. 2022;10:1056817. doi: 10.3389/fpubh.2022.1056817 36544799 PMC9760737

[40] Hall AK, Bernhardt JM, Dodd V, Vollrath MW. The Digital Health Divide: Evaluating Online Health Information Access and Use Among Older Adults. Health Education & Behavior. 2015;42:202–209. doi: 10.1177/1090198114547815 25156311 PMC4405138

[41] Kumari A, Gupta R, Tanwar S. Amalgamation of blockchain and IoT for smart cities underlying 6G communication: A comprehensive review. Computer Communications. 2021;172:102–118.

[42] Guo H, Shang RX, Mi JN. Smart City: Theoretical Origin, Progress and Future Direction——Discovery based on literature mining. E-government. 2022;239:63–73.

[43] Yang QX. How to become a smart city? Decision-Making. 2009;12:54–55.

[44] Hu YF, Zhang YM. Smart governance in the construction of smart city: enabling mechanism and reach path. Journal of Guangxi Normal University (Philosophy and Social Sciences Edition) (2024-01-5) [2024-2-15]. http://kns.cnki.net/kcms/detail/45.1066.C.20240105.1302.002.html.

[45] Pang J, Lei XY, Liu GE. Does Medicare promote health?——Empirical analysis based on the basic medical insurance for urban residents in China. Economic Research; Economic Study. 2013:130–142. https://doi.org/CNKI:SUN:JJYJ.0.2013-04-013.

[46] Shi DQ, Ding H, Wei P, Liu JJ. Whether the construction of smart city can reduce environmental pollution. China’s Industrial Economy. 2018:117–135. doi: 10.19581/j.cnki.ciejournal.2018.06.008

[47] Ye F, Wang Y. Introduction of the dual difference model and its application. Health statistics in China. 2013, 30, 131–133. https://doi.org/CNKI:SUN:ZGWT.0.2013-01-049.

[48] Wang YZ, Sun K, Li L, Lei YL, Wu SM, Jiang Y, et al. The impacts of economic level and air pollution on public health at the micro and macro level. Journal of Cleaner Production. 2022;366:132932.

[49] Bloom DE, Canning D. Population health and economic growth. Health and Growth 2009;24.

[50] Aguila E, Kapteyn A, Smith JP. Effects of income supplementation on health of the poor elderly: The case of Mexico. Proceedings of the National Academy of Sciences. 2015;112:70–75. doi: 10.1073/pnas.1414453112 25535388 PMC4291674

[51] Greiner KA, Li C, Kawachi I, Hunt DC, Ahluwalia JS. The relationships of social participation and community ratings to health and health behaviors in areas with high and low population density. Social Science Medicine. 2004;59:2303–2312. doi: 10.1016/j.socscimed.2004.03.023 15450705

[52] Ferrara L, Chong E, Duryea S. Soap Operas and Fertility: Evidence fromBrazil. American Economic Journal: Applied Economics. 2012;4:1–31.

[53] Chetty R, Looney A, Kroft K. Salience and Taxation: Theory and Evidence. American Economic Review. 2009;99:1145–1177.

[54] Giffmger R, Fertner C, Kramamr H, et al. Smart cities: Ranking of European medium-sized cities. (2007-03-25) [2017-12-12]. http://www.smart-cities. Eu/download/smart-cities-final-report.pdf.

[55] Bolívar MPR, Meijer AJ. Smart governance: Using a literature review and empirical analysis to build a research model. Social Science Computer Review. 2016;34:673–692.

[56] Nicolas C, Kim J, Chi S. Quantifying the dynamic effects of smart city development enablers using structural equation modeling. Sustainable Cities and Society. 2020;53:101916.

[57] Shan W, Xiu C, Ji R. Creating a healthy environment for elderly people in urban public activity space. International Journal of Environmental Research and Public Health. 2020;17:7301. doi: 10.3390/ijerph17197301 33036270 PMC7579163

[58] Farahani M, Subramanian SV, Canning D. Effects ofstate-level public spending on health on the mortality probability inIndia. Health Economics. 2010;19:1361–1376. doi: 10.1002/hec.1557 19937613 PMC3095580

[59] Zeng Y, Gu D, Purser J, Hoenig H, Christakis N. Associations of environmental factors with elderly health and mortality in China. American Journal of Public Health. 2010;100:298–305. doi: 10.2105/AJPH.2008.154971 20019314 PMC2804639

[60] Martínez-Alcalá CI, Pliego-Pastrana P, Rosales-Lagarde A, Lopez-Noguerola JS, Molina-Trinidad EM. Information and communication technologies in the care of the elderly: systematic review of applications aimed at patients with dementia and caregivers. JMIR Rehabilitation and Assistive Technologies. 2016;3:e5226. doi: 10.2196/rehab.5226 28582258 PMC5454565

[61] Kumari A, Tanwar S. Secure data analytics for smart grid systems in a sustainable smart city: Challenges, solutions, and future directions. Sustainable computing: informatics and systems. 2020;28:100427.

[62] Peng Q, Liu ZQ, Wang JD, Hong GW. Study on the coupling and coordinated pattern of green land rate and per capita park green area in built-up areas in China. Modern Urban Studies. 2020:89–96.

[63] Xu ZN, Gao XL, Wang ZQ, Ma Y, Deng Y, Long Y. Evaluation of the green space service level of parks in cities at and above the prefecture level in China: data, models and methods. Geographic Studies. 2019;38:1016–1029.

[64] Hou GY, Hu NN. Analysis of the influence and path improvement of the supply level of health care resources in China. The Chinese Hospital. 2022;26:24–27. doi: 10.19660/j.issn.1671-0592.2022.12.07

[65] Li Z, Liu Y. Research on the spatial distribution pattern and influencing factors of digital economy development in China. IEEE Access. 2021;9:63094–63106.

[66] Zhao T, Zhang Z, Liang SK. Digital economy, entrepreneurial activity and high-quality development——is empirical evidence from Chinese cities. Manage the World. 2020;36:65–76. doi: 10.19744/j.cnki.11-1235/f.2020.0154

[67] Wang C, Liao L, Zhang XM, Lin LT, Chen B. The health and welfare effects of environmental governance: Evidence from China. Environment International. 2024;185:108579. doi: 10.1016/j.envint.2024.108579 38493736

[68] Oueida S, Aloqaily M, Ionescu S. A smart healthcare reward model for resource allocation in smart city. Multimedia Tools and Applications. 2019;78:24573–24594.

[69] Cook DJ, Duncan G, Sprint G, et al. Using smart city technology to make healthcare smarter. Proceedings of the IEEE. 2018;106(4):708–722. doi: 10.1109/JPROC.2017.2787688 29628528 PMC5881605

[70] Hussain A, Wenbi R, Da Silva AL, et al. Health and emergency-care platform for the elderly and disabled people in the Smart City. Journal of Systems and Software. 2015;110:253–263.

[71] Dickinson H, Smith C, Yates S, Tani M. The importance of social supports in education: survey findings from students with disability and their families during COVID-19. Disability & Society. 2021;3:1–23.

[72] Mehta Y, Pai MMM, Mallissery S, et al. Cloud enabled air quality detection, analysis and prediction-a smart city application for smart health[C]//2016 3rd MEC international conference on big data and smart city (ICBDSC). IEEE. 2016:1–7.

[73] You KS, Lee H. The physical, mental, and emotional health of older people who are living alone or with relatives. Archives of Psychiatric Nursing. 2006;20:193–201. doi: 10.1016/j.apnu.2005.12.008 16846780

[74] Zunzunegui MV, Beland F, Otero A. Support from Children, Living Arrangements, Self-Rated Health and Depressive Symptoms of Older People in Spain. International Journal of Epidemiology. 2001;30:1090–1099. doi: 10.1093/ije/30.5.1090 11689528

[75] Ohta S, Nakamoto H, Shinagawa Y, Tanikawa T. A health monitoring system for elderly people living alone. Journal of Telemedicine and Telecare 2002;8:151–156. doi: 10.1177/1357633X0200800305 12097176

[76] Vergados D, Alevizos A, Mariolis A, Caragiozidis M. Intelligent services for assisting independent living of elderly people at home. In Proceedings of the 1st international conference on PErvasive Technologies Related to Assistive Environments. 2008:1–4.

[77] Maki H, Ogawa H, Matsuoka S, Yonezawa Y, Caldwell WM. A daily living activity remote monitoring system for solitary elderly people. IEEE. 2011:5608–5611. doi: 10.1109/IEMBS.2011.6091357 22255611

[78] Heyer A, Granberg RE, Rising KL, Binder AF, Gentsch AT, Handley NR. Medical Oncology Professionals’ Perceptions of Telehealth Video Visits. JAMA Network Open. 2021;4:e2033967. doi: 10.1001/jamanetworkopen.2020.33967 33443581 PMC7809588

[79] Neisi A, Vosoughi M, Shirmardi M, Idani E, Goudarzi G, Hazrati S, et al. Concentration of air pollutants as toxic matter in urban and rural areas of Ahvaz. Toxin Reviews. 2018;37:243–250.

[80] Currie J. Inequality at Birth:Some Causes and Consequences. American Economic Review. 2011;101:1–22.

[81] Mazzeo RS, Tanaka H. Exercise prescription for the elderly: current recommendations. Sports Medicine. 2001;31:809–818. doi: 10.2165/00007256-200131110-00003 11583105

[82] Krenichyn K. ‘The only place to go and be in the city’: women talk about exercise, being outdoors, and the meanings of a large urban park. Health place. 2006;12:631–643. doi: 10.1016/j.healthplace.2005.08.015 16188484

[83] Yasunaga A, Tokunaga M. The relationships among exercise behavior, functional ADL, and psychological health in the elderly. Journal of Physiological Anthropology and Applied Human Science. 2001;20:339–343. doi: 10.2114/jpa.20.339 11840686

[84] Gaikwad A, Shinde K. Use ofparks by older persons and perceivedhealth benefits:A developing country context. Cities. 2019;84:134–142.

[85] Frankel JE, Bean JF, Frontera WR. Exercise in the elderly: research and clinical practice. Clinics in Geriatric Medicine. 2006;22:239–256. doi: 10.1016/j.cger.2005.12.002 16627076

[86] Orsega-Smith E, Mowen AJ, Payne LL, Godbey G. The interaction of stress and park use on psychophysiological health in older adults. Journal of Leisure Research. 2004;36:232–256.

[87] The state council of the people’s republic of China. Guiding Opinions on Promoting the Healthy Development of Smart Cities. (2014-08-27) [2024-2-15]. https://www.gov.cn/gongbao/content/2015/content_2806019.htm.

[88] Gertler PJ, Patrinos HA, Rubio-Codina M. Empowering parents to improve education:Evidence from rural Mexico. Journal of Development Economics. 2012;99:68–79.

[89] Ramírez-Moreno MA, Keshtkar S, Padilla-Reyes DA, Ramos-López E, García-Martínez M, Hernández-Luna MC, et al. Sensors for sustainable smart cities: A review. Applied Sciences. 2021;11:8198.

[90] Ko FWS, Hui DSC. Air pollution and chronic obstructive pulmonary disease. Respirology. 2012;17:395–401. doi: 10.1111/j.1440-1843.2011.02112.x 22142380

[91] Guarnieri M, Balmes JR. Outdoor air pollution and asthma. The Lancet. 2014;383:1581–1592. doi: 10.1016/S0140-6736(14)60617-6 24792855 PMC4465283

[92] Pirozzi CS, Jones BE, VanDerslice JA, Zhang Y, Paine R III, Dean NC. Short-term air pollution and incident pneumonia. A case-crossover study. Annals of the American Thoracic Society. 2018;15:449–459. doi: 10.1513/AnnalsATS.201706-495OC 29283681

[93] Abolhasani E, Hachinski V, Ghazaleh N, Azarpazhooh MR, Mokhber N, Martin J. Air pollution and incidence of dementia: A systematic review and meta-analysis. Neurology. 2023;100:e242–e254. doi: 10.1212/WNL.0000000000201419 36288998

[94] Wang J, Ma T, Ma D, Li H, Hua L, He Q, et al. The impact of air pollution on neurodegenerative diseases. Therapeutic Drug Monitoring. 2021;43:69–78. doi: 10.1097/FTD.0000000000000818 33009291

[95] Deen MJ. Information and communications technologies for elderly ubiquitous healthcare in a smart home. Personal and Ubiquitous Computing. 2015;19:573–599.

[96] Goodall K, Ward P, Newman L. Use of information and communication technology to provide health information: what do older migrants know, and what do they need to know? Quality in Primary Care. 2010;18:27–32. 20359410

[97] Goswami S, Köbler F, Leimeister JM, Krcmar H. Using online social networking to enhance social connectedness and social support for the elderly. 2010.

[98] Sum S, Mathews RM, Hughes I, Campbell A. Internet use and loneliness in older adults. Cyberpsychology Behavior and Social Networking. 2008;11:208–211. doi: 10.1089/cpb.2007.0010 18422415

